# Metabolomics as an emerging tool to study plant–microbe interactions

**DOI:** 10.1042/ETLS20210262

**Published:** 2022-02-22

**Authors:** Sneha Gupta, Martino Schillaci, Ute Roessner

**Affiliations:** 1 School of BioSciences, University of Melbourne, Parkville, VIC, Australia; 2 Consiglio Nazionale Delle Ricerche-Istituto per la Protezione Sostenibile Delle Piante, Strada delle Cacce 73, 10135 Torino, Italy

**Keywords:** biochemical techniques and resources, lipidomics, metabolomics, plant–microbe interactions

## Abstract

In natural environments, interaction between plant roots and microorganisms are common. These interactions between microbial species and plants inhabited by them are being studied using various techniques. Metabolomics research based on mass spectrometric techniques is one of the crucial approaches that underpins system biology and relies on precision instrument analysis. In the last decade, this emerging field has received extensive attention. It provides a qualitative and quantitative approach for determining the mechanisms of symbiosis of bacteria and fungi with plants and also helps to elucidate the tolerance mechanisms of host plants against various abiotic stresses. However, this -omics application and its tools in plant–microbe interaction studies is still underutilized compared with genomic and transcriptomic methods. Therefore, it is crucial to bring this field forward to bear on the study of plant resistance and susceptibility. This review describes the current status of methods and progress in metabolomics applications for plant–microbe interaction studies discussing current challenges and future prospects.

## Introduction

Metabolomics aims to qualitatively and quantitatively analyse the metabolites of living systems and their dynamic responses to changes in the environment [1–3]. Analysis, detection and identification of metabolites are the core of any metabolomics approach. The field of metabolomics originated from metabolite profiling in the 1970s [4]. Fiehn et al. [5] proposed the term ‘metabolomics' and defined it as a ‘comprehensive and quantitative analysis of all metabolites in a biological system'. Metabolomics focuses on all small molecule components and the fluctuations in individual cells, cell types, tissue types or organs and is often used to study plant and microbial systems. Today, metabolomics is a growing field among omics sciences that is mainly concerned with high-throughput snapshots of metabolomes [6].

**Figure 1.**
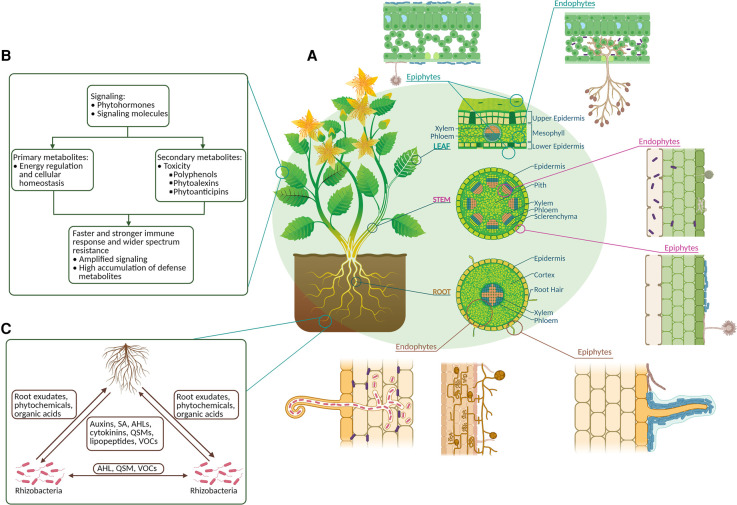
Diagram showing possible sites of interactions between plants and microbiota (A) taken from Walker et al. [10] and compounds secreted by the microbes and metabolite profiles present in leaves (B) and root exudates (C). (A) Blue rods, bacterial epiphytes; dark purple rods, bacterial endophytes; red rods, root nodule bacteria; fungi shown in brown and grey. Not to scale. Abbreviations: volatile organic compounds (VOCs), quorum sensing molecules (QSM), N-acyl homoserine lactones (AHL), Salicylic acid (SA).

In the natural environment, plants are inhabited by a large number of microbiota that include fungi, bacteria, actinomycetes, algae, and protozoa [7]. Hence, it is difficult to generalize plant-physiology due to this diversity. Among these organisms, the majority of these are bacteria [8]. Plant growth promoting bacteria (PGPB) live mostly inside the rhizosphere and/or roots of plants and can be extremely beneficial for their growth and development. Similarly, fungi play an important role in natural and agricultural ecosystems. They act as important decomposers and recyclers of organic materials and they can interact with the belowground or with aboveground plant tissues [9]. These interactions between plants and fungi are complex and the outcomes are diverse, ranging from parasitism to mutualism. Figure 1A summarizes the sites where these interactions occur between microbiota and plant tissue.

To analyse the interactions between various species, the application of metabolomics not only provides a comprehensive picture of metabolic pathways that are involved but also explains the underlying mechanisms of host and microbial interactions. Figure 1B,C illustrates examples of compounds secreted during the beneficial relationships between plants and associated microbiota. Interactions between fungi and their hosts is an intriguing field comprising of the interactions of fungal species with plant, insect, animal or human hosts. In this review, we aim to summarize the advantages of metabolomics analysis platforms and their applications in investigating the interactions of different species while focusing on plant–bacterial and plant–fungal interactions.

## Metabolomics methods for plant–bacterial/fungal interactions

**Figure 2.**
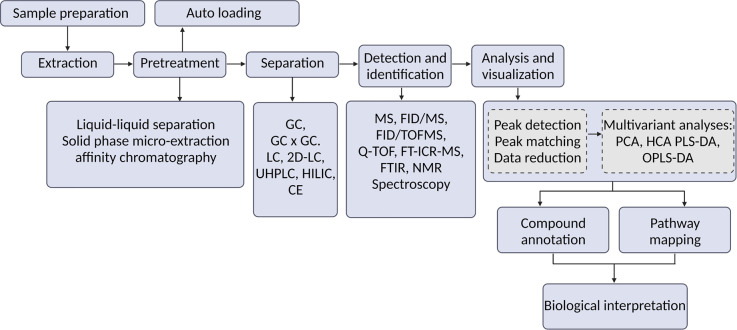
Metabolomics analysis flow for plant–microbe interaction research. GC, gas chromatography; LC, liquid chromatography; UHPLC, Ultra-High-Performance Liquid Chromatography; HILIC, hydrophilic interaction liquid chromatography; CE, capillary electrophoresis; FID/MS, flame ionization detector mass spectrometry; FID/TOFMS, flame ionization detector time of flight mass spectrometry; Q-TOF, quadrupole time of flight; FT-ICR-MS, Fourier transform ion cyclotron resonance mass spectrum; FTIR, fourier transform infrared; NMR, nuclear magnetic resonance; PCA, principal component analysis; HCA, hierarchical cluster analysis; PLS-DA, partial least squares discriminant analysis; OPLS-DA, orthogonal partial least squares discriminant analysis; HMDB: human metabolome database; KEGG, Kyoto encyclopedia of genes and genomes. Figure adapted from Chen et al. [4].

Metabolomics is a well-known technique for studying plant–microbe interactions. Several studies have been reported on important biotic interactions in plants, especially with mycorrhiza, PGPB, and many forms of filamentous fungi, including different Trichoderma strains. Numerous theoretical viewpoints have been shared on the advantageous relations such as nutrient intake, receptor recognition, and positive effect on growth and development. Metabolomics has played a key role in elucidating the connection and the differentiation between disease or symbiosis. The communications between Arabidopsis, PGPB and other microbes have been investigated together showing an effective illustration of the integration of different types of omics data [11]. Although there are ample of studies reported using metabolomics techniques, research in this field is still under rapid development with constantly evolving methodologies [12]. Figure 2 shows the compilation of techniques mentioned above and more, including experimental design methods, sample preparation, data acquisition, data processing, and biological interpretation relevant to metabolomics of plant–microbe interaction research.

## Omics era and recent plant microbiome research expansion

Plants and microbes often communicate in the rhizosphere where plant roots secrete exudates. The rhizosphere communities i.e. the microorganisms surrounding the root regions are influenced by the chemical composition of these root exudates [13]. For example, Zhang et al. [14] showed that the exudates released by cucumber roots attracted *Bacillus amyloliquefaciens* SQR9, in which citric acid was identified, causing biofilm formation [14]. The host plant and the metabolism of microbes are affected by several bacterial genes such as nodulation genes. These genes are responsible for producing Nod factors during nodulation of roots [15]. Metabolomics has been employed for studying the chemical exudation process from grassroots during development, showing its effect on the assembly of the rhizosphere community [16]. Organic acids were the primary exuded metabolites consumed by the bacterial enrichment in the substrate [17]. Flavonoids are another class of compounds that are found in root exudates, which induce bacterial nod genes and initiate nodule formation in the root [18]. Another study by Negrel et al. [19] identified a number of lipid markers of *Plasmopara viticola* inoculation in grapevines through untargeted metabolomics studies.

Metabolomics has been useful in showing that the relationship among plants and PGPB can be quite specific. An example of this is given by Chamam et al. [20] where two Azospirillum strains improved the growth of two rice (*Oryza sativa*) cultivars, but affected their metabolome differently. Walker et al. [21] combined LC–MS and nuclear magnetic resonance (NMR) to study the metabolism of two maize cultivars inoculated with three different *Azospirillum* strains. The results showed that these bacteria were strain-dependent showing effects only on the secondary metabolism of inoculated plants without affecting the primary metabolism involving the physiological functions such as plant growth and development. Another metabolomic study on the interaction between different maize genotypes and two nitrogen fixing PGPB species or their mutants with impaired nitrogenase activity allowed the identification of plant compounds affected by bacterial nitrogen fixation [22].

A parallel protocol on the relationship among plants and symbionts to be specific has been shown extensively for arbuscular mycorrhiza (AM) where five distinct plant species were inhabited with a single AM and the metabolomic profiling indicated remarkably divergent and species-specific metabolic changes regardless of a retained ‘core metabolome' [23]. In another study, UHPLC combined with time-of-flight mass spectrometer Q-TOF/MS was used to detect putative mycorrhiza-associated metabolites in *Medicago truncatula* colonized roots with the AM fungus *Rhizophagus irregularis*. The study detected 71 mycorrhiza-associated metabolites with at least 10-fold more in mycorrhizal roots as compared with non-colonized roots [24]. Like investigating mycorrhizal fungi, metabolomics has been also extensively used to study endophytic fungi in many plant tissues.

Recently, Valette et al. [25] combined metabolite analysis using Ultra High Performance Liquid Chromatography-Diode Array Detector hyphenated to high resolution electrospray ionization time-of-flight mass spectrometer (UHPLC-DAD/ESI-QTOF) with gene expression data to obtain high resolution datasets of secondary metabolites affected by the inoculation with different PGPB species in rice roots and to obtain further insights into the role of specific affected metabolites. In another study by Mhlongo et al. [26], UHPLC coupled to a triple quadrupole mass spectrometer (UHPLC-QqQ-MS) techniques provided new insights into the plant defense mechanisms involved in the early interactions between tomato and various PGPB species.

Matrix-assisted laser desorption ionization mass spectrometric imaging (MALDI-MSI) is another technique that is recently used for gaining the spatial information of metabolites in inoculated roots and root nodules. Recently, the metabolite profiles of roots and nodules of *Medicago truncatula* associated with Sinorhizobium meliloti during nitrogen fixation was studied using MALDI-MSI [27]. This study detected various amino acids, organic acids, sugars, lipids and flavonoids, along with molecular ion images obtained from nitrogen-fixing and non-fixing nodules. MALDI-MSI has been employed to identify and solve the spatiotemporal production of antimicrobial compounds (surfactins, iturins, pliplastatin, streptorubin and fengycin) released by root-colonizing Bacillus (e.g. *B. subtilis* and *B. amyloliquefaciens*) [28,29]. Furthermore, MALDI MSI coupled with MS/MS analysis was used to study the spatiotemporal changes and identify antibiotic compounds released by roots of *Solanum lycopersicum* biofilmed with *B. amyloliquefaciens*. The results from this study identified a new variant of surfactins released at later time points post-inoculation [30].

In a study by Cao et al. [31], the metabolic effect of endophytic *Neotyphodium lolii* and its host perennial ryegrass (*Lolium perenne*) have been studied by direct infusion mass spectrometry (MS) in immature leaves, sheaths and blades, with data obtained from MS^2^ and MS^3^ product ion spectra. The results showed changes in the metabolome in inoculated plants, with key compounds such as mannitol, peramine and perloline. Another study by Waqas et al. (2012) [32] showed the production of hormone substances, such as auxin, gibberellin (GA), and cytokinin from endophytic fungi which directly promoted rice plant growth. Recently, the technique of UHPLC-Q-TOF/MS for the rapid identification and profiling of several structurally similar bioactive shikonins from the root periderm tissues of Australian annual weed *Echium plantagineum* and Australian perennial weed *E. vulgare* has been used by Skoneczny et al. [33]. The results showed that shikonin levels was 2.5 times higher in *E. vulgare* extracts as compared with *E. plantagineum*.

Endophytic fungi are known to produce antagonistic substances that not only help plants resist pathogenic microorganisms but can also indirectly induce and activate the plant defense system. This helps in improving the plant defense ability and adaptation to diseases and insect pests. The antagonistic substances produced by endophytic fungi mainly include small-molecular-weight active substances and antimicrobial peptides. In a recent study by Liu et al. [34], NMR and MS spectrum data were used to identify a strain of *Aspergillus racemose* from mangrove plants in Hainan, which produces the metabolites 22-epi-aflaquinolone B (used against wheat total erosion bacteria) and 14-epi-isochaetominine C, used against wheat scab in agricultural bacterial diseases.

In one of the few metabolomic studies on tripartite associations (plant–bacteria–fungi), Roupael et al. [35] applied a mixture of PGPB and beneficial fungi with or without a protein-based biostimulant to maize plants. UHPLC analysis of shoot and root samples displayed a distinct metabolic response in each of the treatments, particularly showing an effect on secondary metabolites.

There is growing interest in linking metabolomics with lipidomics [36]. This integrated approach could contribute to a better understanding of metabolism which metabolomics alone would otherwise lack in defining [37]. Several studies have been reported using this integrated approach to understand the rearrangements of metabolites and lipids during plant–microbe interactions. For example, metabolomics techniques such as untargeted GC–MS and untargeted QqTOF LC–MS were used to compare the root metabolic and lipid profiles of inoculated and non-inoculated *Brachypodium distachyon* Bd21–3 plants with *Azospirillum brasilense* Sp245 grown at low temperatures and supplied with insufficient phosphorus. It has been proposed that the nutritional status of the plant influenced the interaction of the plant with Azospirillum: bacteria were sensed as pathogens despite sufficient phosphorus in plants, however, the interaction became beneficial for the plants as their phosphorus levels decreased [38]. Another study by Gupta et al. [39] used GC–MS to analyse polar metabolites and LC–MS to analyse lipids in roots of two barley cultivars (with contrasting salinity tolerance) during the early stages of interaction with *Trichoderma harzianum* T-22. They found that many of the metabolic changes in the inoculated salt treated sensitive cultivar show similar responses as the uninoculated tolerant cultivar, while a number of metabolites were changed in both cultivars following fungal inoculation only.

## Plant–microbe interactions metabolomics experimental setup

When research is mainly focused on root interactions with PGPB or fungi, seed preparation and inoculation play a major role in the successful establishment of the plant–microbe relationship. To isolate the effects of microbial inoculation on plant metabolism, it is essential to carefully sterilize the seed surface using appropriate protocols. While this step will not eliminate the possible seed endophytes vertically transmitted from the parental generation, it will surely decrease the microbial population. Seeds can then be germinated and transplanted to the growth substrate or directly planted. In the first case, seeds can be inoculated prior to the transplant by dipping their roots in a bacterial solution containing the inoculum at the desired strength. Alternatively, plants can also be inoculated in their substrate, by pouring the desired volume of bacterial solution on them. Similar protocols can be followed for seeds intended to be inoculated with fungus where seeds can either be submerged in a solution containing fungal spores or alternatively roots can be inoculated by pouring the desired volume with a specific number of spores. If the metabolomic study is the first to characterize the interaction between a specific plant and a PGP microorganism, it may be preferable to grow plants in a sterilized substrate and in a controlled environment (growth chamber, greenhouse), in order to minimize the noise that would come from a more complex setup. The hypotheses obtained from ‘reductionist' experiments can then be tested in more natural environments, where conditions change continuously, and plants interact also with native microbiota. At harvest, plant metabolism should be quenched as soon as possible by shock-freezing the plant tissue.

## Prospects and challenges

To attain the goal of holistic analyses of the metabolome, a wide range of chemometrics methods, chemistries, advanced analytical instrumentation and novel computational approaches providing high degrees of sensitivity and reproducibility, are required in metabolomics. In contrast with other -omics methodologies, metabolomics encounters several unique challenges making the field particularly demanding. These challenges arise especially from the inherent characteristics of the metabolome such as: (i) being highly dynamic where metabolome is changing continuously at different rates, (ii) chemically diverse nature of metabolites with different physicochemical properties, biological functions and highly diverse and dynamic stereo chemistries, (iii) wide range of metabolite levels and the bio-complexity of living systems involving several biological cycles and compartmentalization at the organism and cellular level) [40,41,42,43]. These challenges are more complex when plants constituting an enormous treasure of microbiome are explored. There has been a tremendous progress in understanding the plant microbiome. However, further understanding of the processes is still required that will help understand the microbiome community formation and function in plants.

- The early stages of contact between plants and microorganisms retain particular interest, as they usually define whether the latter will be perceived as beneficial or pathogenic [44]. Recent studies have also shown that the relationship between plants and microorganisms can change with the worsening of the environmental conditions [45], and determining what mechanisms take place in the interplay at those stages is of great importance. Future studies should combine frequent analysis of inoculated plants with newly developed methods such as MALDI-MSI, which allow to determine the location of the detected metabolites in the analyzed tissues. By doing so, they could precisely resolve into time and tissue distribution of the compounds being involved in the interaction, in order to better understand its underlying mechanisms.
- The metabolomic analysis of plant tissues inoculated with PGP bacteria seldom allows to determine whether the detected metabolites were synthesized by the plant or by the microorganisms [46], and this task is still difficult to achieve. The sole comparison with the metabolome of plants and microorganisms grown in axenic culture can provide some useful information, but does not give the full picture, as there still can be compounds produced by an organism only when interacting with another. Integrating metabolomics with the analysis of the transcriptome of plants and microorganisms alone and interacting with each other can help in this task by ascribing the various metabolites to the organism which also show an increased expression of the genes related to their metabolism [38].
- As mentioned above, there has been little or no effort made in differentiating microbial metabolites from plant metabolites as the microbes are not removed from the metabolite extraction [47,48,49,50]. This becomes a problem especially, when species specific databases are available, as there can be cross-contamination between the plant and the microbial metabolome which is a serious issue. To solve this problem, culturing microbial cells in stable isotope media can be a powerful way to trace the origin of biomolecules. Stable isotope labeling can be applied for reference metabolite labeling (e.g. for accurate quantification), metabolic flux analysis and identification of metabolites in different organisms [51,52,53,54]. For example, a study performed by Pang et al. [55] used targeted metabolomic analysis on *Arabidopsis thaliana* epidermal peels with guard cells infected witha plant bacterial pathogen *Pseudomonas syringae* pv. tomato (*Pst*) DC3000 labeled with heavy isotopes. The results suggested that *Pst* DC3000 infection changed plant metabolites, including signaling and primary metabolites.
- High resolution mass spectrometry (HRMS) is another powerful technique that has undergone an exciting phase of technological evolution with application in plant–microbe interactions studies [56,57]. The HRMS instrumentation has increased mass resolution that help to distinguish between isotopic distributions and generate fragmentation patterns. This improves the accuracy of predicting the chemical formula and identifying compounds through library matching. However, HRMS instrumentation does not replace standard low-resolution mass spectrometers used in several research laboratories. For example, for nontargeted analyses where HRMS is advantageous, high resolution is not necessary for basic applications. HRMS generates highly complex data in large quantities, which is not necessary for routine, targeted analyses as these can be processed using low-resolution mass spectrometry. For such targeted analyses of a limited subset of compounds, single Da resolution methods can be used for detection and quantification [58].
- Metabolomics is an essential link between transcriptomics and phenomics, potentially allowing to identify the genes responsible for the phenotype differences observed in the organisms following different treatments. If the genome of such organisms has already been sequenced and annotated, the genes linked to the different metabolites could then be selected for further studies. In the case of plants inoculated with PGP microorganisms, metabolites responsible for an improved phenotype could thus be linked to the respective genes and then be researched in varieties that express them at higher levels. Alternatively, those genes could be edited to be constitutionally expressed in already profitable varieties, in order to further increase their fitness.
- Normally, metabolomic studies of plant–microbe interactions are first performed in controlled environments (growth chambers, greenhouses), which allow to isolate the different inputs in the system and clearly define the relations among them. If the final goal of metabolomic studies is to produce knowledge to improve future agriculture, the hypotheses drawn from such experiments should be validated in progressively less controlled environments, where plants are subjected to environmental fluctuations.
- In the past years, most of the metabolomic studies were performed on the response of the model plant Arabidopsis to external stimuli [59]. In the upcoming years, it will be necessary to extend the metabolomic studies to agriculturally relevant crops, which often differ strongly from the most studied model plant species.

## Summary

- Metabolomics is a data-driven, hypothesis-generating scientific approach which aims to detect and quantify 1000s of compounds per analysis. It provides a suitable approach to study complex biological interactions within the rhizosphere and reciprocal responses between plants and organisms.
- Metabolomics is an emerging field in the plant sciences, however its application to study beneficial plant microbe interactions is lagging compared with other omics approaches, providing many opportunities to broaden our understanding of the underlaying mechanisms of beneficial plant microbe interactions
- Application of metabolomics to study plant microbe interactions comes with several challenges, such as the differentiation of the origin of metabolites analysed, uncovering the metabolic complexity of two or more organisms interacting and linking metabolome information with other omics data such as transcriptomics, proteomics or phenomics.

## Open Access

Open access for this article was enabled by the participation of University of Melbourne in an all-inclusive *Read & Publish* agreement with Portland Press and the Biochemical Society under a transformative agreement with CAUL.

## Abbreviations

AM: arbuscular mycorrhiza
HRMS: high resolution mass spectrometry
NMR: nuclear magnetic resonance
PGPB: plant growth promoting bacteria

## References

[1] Fiehn, O. (2001) Combining genomics, metabolome analysis, and biochemical modelling to understand metabolic networks. Comp. Funct. Genom. 2, 155–168 10.1002/cfg.82 PMC2447208 18628911

[2] Oliver, S.G., Winson, M.K., Kell, D.B. and Baganz, F. (1998) Systematic functional analysis of the yeast genome. Trends Biotechnol. 16, 373–378 10.1016/S0167-7799(98)01214-1 9744112

[3] Ryan, D. and Robards, K. (2006) Metabolomics: the greatest omics of them all? Anal. Chem. 78, 7954–7958 10.1021/ac0614341 17134127

[4] Chen, F., Ma, R. and Chen, X.L. (2019) Advances of metabolomics in fungal pathogen–plant interactions. Metabolites 9, 169 10.3390/metabo9080169 PMC6724083 31443304

[5] Fiehn, O., Kopka, J., Dörmann, P., Altmann, T., Trethewey, R.N. and Willmitzer, L. (2000) Metabolite profiling for plant functional genomics. Nat. Biotechnol. 18, 1157–1161 10.1038/81137 11062433

[6] Shafi, A., Zahoor, I. and Habib, H. (2021) Omics Technologies to Unravel Plant-Microbe Interactions. In Plant-Microbe Dynamics: Recent Advances for Sustainable Agriculture (Pirzadah, T.B., Malik, B. and Hakeem, K.R. eds), pp. 201–220, CRC Press, Boca Raton, Florida, USA

[7] Pirzadah, T.B., Malik, B. and Hakeem, K.R. (2021) Plant-Microbe Dynamics: Recent Advances for Sustainable Agriculture, CRC press, London

[8] Glick, B.R. (2012) Plant growth-promoting bacteria: mechanisms and applications. Scientifica 2012, 963401 10.6064/2012/963401 24278762 PMC3820493

[9] Zeilinger, S., Gupta, V.K., Dahms, T.E., Silva, R.N., Singh, H.B., Upadhyay, R.S. et al. (2016) Friends or foes? Emerging insights from fungal interactions with plants. FEMS Microbiol. Rev. 40, 182–207 10.1093/femsre/fuv045 26591004 PMC4778271

[10] Walker, R., Otto-Pille, C., Gupta, S., Schillaci, M. and Roessner, U. (2020) Current perspectives and applications in plant probiotics. Microbiology 41, 95–99 10.1071/MA20024

[11] van de Mortel, J.E., de Vos, R.C., Dekkers, E., Pineda, A., Guillod, L., Bouwmeester, K. et al. (2012) Metabolic and transcriptomic changes induced in Arabidopsis by the rhizobacterium *pseudomonas fluorescens* SS101. Plant Physiol. 60, 2173–2188 10.1104/pp.112.207324 PMC3510139 23073694

[12] Li, N., Peng Song, Y., Tang, H. and Wang, Y. (2016) Recent developments in sample preparation and data pre-treatment in metabonomics research. Arch. Biochem. 589, 4–9 10.1016/j.abb.2015.08.024 26342458

[13] Sasse, J., Martinoia, E. and Northen, T. (2018) Feed your friends: do plant exudates shape the root microbiome? Trends Plant Sci. 23, 25–41 10.1016/j.tplants.2017.09.003 29050989

[14] Zhang, N., Wang, D., Liu, Y., Li, S., Shen, Q., Zhang, R. et al. (2014) Effects of different plant root exudates and their organic acid components on chemotaxis, biofilm formation and colonization by beneficial rhizosphere-associated bacterial strains. Plant Soil. 374, 689–700 10.1007/s11104-013-1915-6

[15] Sharma, M., Sudheer, S., Usmani, Z., Rani, R. and Gupta, P. (2020) Deciphering the omics of plant-microbe interaction: perspectives and new insights. Curr. Genom. 21, 343–362 10.2174/1389202921999200515140420 PMC7536805 33093798

[16] van Dam, N.M. and Bouwmeester, H.J. (2016) Metabolomics in the rhizosphere: tapping into belowground chemical communication. Trends Plant Sci. 21, 256–265 10.1016/j.tplants.2016.01.008 26832948

[17] Zhalnina, K., Louie, K.B., Hao, Z., Mansoori, N., da Rocha, U.N., Shi, S. et al. (2018) Dynamic root exudate chemistry and microbial substrate preferences drive patterns in rhizosphere microbial community assembly. Nat. Microbiol. 3, 470–480 10.1038/s41564-018-0129-3 29556109

[18] Mhlongo, M.I., Piater, L.A., Madala, N.E., Labuschagne, N. and Dubery, I.A. (2018) The chemistry of plant–microbe interactions in the rhizosphere and the potential for metabolomics to reveal signaling related to defense priming and induced systemic resistance. Front. Plant Sci. 9, 112 10.3389/fpls.2018.00112 29479360 PMC5811519

[19] Negrel, L., Halter, D., Wiedemann-Merdinoglu, S., Rustenholz, C., Merdinoglu, D., Hugueney, P. et al. (2018) Identification of lipid markers of *Plasmopara viticola* infection in grapevine using a non-targeted metabolomic approach. Front. Plant Sci. 9, 360 10.3389/fpls.2018.00360 29619037 PMC5871909

[20] Chamam, A., Sanguin, H., Bellvert, F., Meiffren, G., Comte, G., Wisniewski-Dyé, F. et al. (2013) Plant secondary metabolite profiling evidences strain-dependent effect in the Azospirillum–*Oryza sativa* association. Phytochemistry 87, 65–77 10.1016/j.phytochem.2012.11.009 23266268

[21] Walker, V., Bertrand, C., Bellvert, F., Moënne-Loccoz, Y., Bally, R. and Comte, G. (2011) Host plant secondary metabolite profiling shows a complex, strain-dependent response of maize to plant growth-promoting rhizobacteria of the genus Azospirillum. New Phytol. 189, 494–506 10.1111/j.1469-8137.2010.03484.x 20946131

[22] Brusamarello-Santos, L.C., Gilard, F., Brulé, L., Quilleré, I., Gourion, B., Ratet, P. et al. (2017) Metabolic profiling of two maize (*Zea mays* L.) inbred lines inoculated with the nitrogen fixing plant-interacting bacteria *Herbaspirillum seropedicae* and *Azospirillum brasilense*. PLoS ONE 12, e0174576 10.1371/journal.pone.0174576 28362815 PMC5375134

[23] Schweiger, R., Heise, A.M., Persicke, M. and Müller, C.J. (2014) Interactions between the jasmonic and salicylic acid pathway modulate the plant metabolome and affect herbivores of different feeding types. Plant Cell Environ. 37, 1574–1585 10.1111/pce.12257 24372400

[24] Laparre, J., Malbreil, M., Letisse, F., Portais, J.C., Roux, C., Bécard, G. et al. (2014) Combining metabolomics and gene expression analysis reveals that propionyl-and butyryl-carnitines are involved in late stages of arbuscular mycorrhizal symbiosis. Mol. Plant 7, 554–566 10.1093/mp/sst136 24121293

[25] Valette, M., Rey, M., Gerin, F., Comte, G. and Wisniewski-Dyé, F. (2020) A common metabolomic signature is observed upon inoculation of rice roots with various rhizobacteria. J. Integr. Plant Biol. 62, 228–246 10.1111/jipb.12810 30920733

[26] Mhlongo, M.I., Piater, L.A., Steenkamp, P.A., Labuschagne, N. and Dubery, I.A. (2020) Metabolic profiling of PGPR-treated tomato plants reveal priming-related adaptations of secondary metabolites and aromatic amino acids. Metabolites 10, 210 10.3390/metabo10050210 PMC7281251 32443694

[27] Ye, H., Gemperline, E., Venkateshwaran, M., Chen, R., Delaux, P.M., Howes-Podoll, M. et al. (2013) MALDI mass spectrometry-assisted molecular imaging of metabolites during nitrogen fixation in the *Medicago truncatula–Sinorhizobium meliloti* symbiosis. Plant J. 75, 130–145 10.1111/tpj.12191 23551619

[28] Moree, W.J., Yang, J.Y., Zhao, X., Liu, W.T., Aparicio, M., Atencio, L. et al. (2013) Imaging mass spectrometry of a coral microbe interaction with fungi. J. Chem. Ecol. 39, 1045–1054 10.1007/s10886-013-0320-1 23881443 PMC3839821

[29] Pathak, K.V. and Keharia, H. (2013) Characterization of fungal antagonistic bacilli isolated from aerial roots of banyan (*Ficus benghalensis*) using intact-cell MALDI-TOF mass spectrometry (ICMS). J. Appl. Microbiol. 114, 1300–1310 10.1111/jam.12161 23387377

[30] Debois, D., Jourdan, E., Smargiasso, N., Thonart, P., De Pauw, E. and Ongena, M. (2014) Spatiotemporal monitoring of the antibiome secreted by Bacillus biofilms on plant roots using MALDI mass spectrometry imaging. Anal. Chem. 86, 4431–4438 10.1021/ac500290s 24712753

[31] Cao, M., Koulman, A., Johnson, L.J., Lane, G.A. and Rasmussen, S. (2008) Advanced data-mining strategies for the analysis of direct-infusion ion trap mass spectrometry data from the association of perennial ryegrass with its endophytic fungus, *Neotyphodium lolii*. Plant Physiol. 146, 1501–1514 10.1104/pp.107.112458 18287492 PMC2287329

[32] Waqas, M., Khan, A.L., Kamran, M., Hamayun, M., Kang, S.M., Kim, Y.H. et al. (2012) Endophytic fungi produce gibberellins and indoleacetic acid and promotes host-plant growth during stress. Molecules 17, 10754–10773 10.3390/molecules170910754 22960869 PMC6268353

[33] Skoneczny, D., Weston, P.A., Zhu, X., Gurr, G.M., Callaway, R.M., Barrow, R.A. et al. (2017) Metabolic profiling and identification of shikonins in root periderm of two invasive *echium* spp. weeds in Australia. Molecules 22, 330 10.3390/molecules22020330 PMC6155885 28230806

[34] Liu, R., Bao, Z.X., Zhao, P.J. and Li, G.H. (2021) Advances in the study of metabolomics and metabolites in some species interactions. Molecules 26, 3311 10.3390/molecules26113311 34072976 PMC8197931

[35] Rouphael, Y., Lucini, L., Miras-Moreno, B., Colla, G., Bonini, P. and Cardarelli, M. (2020) Metabolomic responses of maize shoots and roots elicited by combinatorial seed treatments with microbial and non-microbial biostimulants. Front. Microbiol. 11, 664 10.3389/fmicb.2020.00664 32435233 PMC7218175

[36] Gallart-Ayala, H., Teav, T. and Ivanisevic, J. (2020) Metabolomics meets lipidomics: assessing the small molecule component of metabolism. Bioassays 42, 2000052 10.1002/bies.202000052 33230910

[37] Johnson, C.H., Ivanisevic, J. and Siuzdak, G.J. (2016) Metabolomics: beyond biomarkers and towards mechanisms. Nat. Rev. Mol. 17, 451–459 10.1038/nrm.2016.25 PMC5729912 26979502

[38] Schillaci, M., Kehelpannala, C., Martinez-Seidel, F., Smith, P., Arsova, B., Watt, M. et al. (2021) The metabolic response of brachypodium roots to the interaction with beneficial bacteria is affected by the plant nutritional status. Metabolites 11, 358 10.3390/metabo11060358 34205012 PMC8228974

[39] Gupta, S., Smith, P., Boughton, B.A., Rupasinghe, T.W., Natera, S.H. and Roessner, U. (2021) Inoculation of barley with *Trichoderma harzianum* T-22 modifies lipids and metabolites to improve salt tolerance. J. Exp. Bot. 72, 7229–7246 10.1093/jxb/erab335 34279634

[40] Beisken, S., Eiden, M. and Salek, R.M. (2015) Getting the right answers: understanding metabolomics challenges. Expert Rev. Mol. Diagn. 15, 97–109 10.1586/14737159.2015.974562 25354566

[41] Kell, D.B., Brown, M., Davey, H.M., Dunn, W.B., Spasic, I. and Oliver, S.G. (2005) Metabolic footprinting and systems biology: the medium is the message. Nat. Rev. Microbiol. 3, 557–565 10.1038/nrmicro1177 15953932

[42] Hall, R.D. (2011) Plant metabolomics in a nutshell: potential and future challenges. In Biology of Plant Metabolomics (Wiley Blackwell, R.D. ed.), Hall, Oxford, pp. 1–24

[43] Heinig, U., Gutensohn, M., Dudareva, N. and Aharoni, A. (2013) The challenges of cellular compartmentalization in plant metabolic engineering. Curr. Opin. Biotechnol. 24, 239–246 10.1016/j.copbio.2012.11.006 23246154

[44] Macabuhay, A., Arsova, B., Walker, R., Johnson, A., Watt, M. and Roessner, U. (2021) Modulators or facilitators? Roles of lipids in plant root–microbe interactions. Trends Plant Sci. 27, 180–190 10.1016/j.tplants.2021.08.004 34620547

[45] Hardoim, P.R., Van Overbeek, L.S., Berg, G., Pirttilä, A.M., Compant, S., Campisano, A. et al. (2015) The hidden world within plants: ecological and evolutionary considerations for defining functioning of microbial endophytes. Microbiol. Mol. Biol. Rev. 79, 293–320 10.1128/MMBR.00050-14 26136581 PMC4488371

[46] Ludwig-Müller, J. (2015) Plants and endophytes: equal partners in secondary metabolite production? Biotechnol. Lett. 37, 1325–1334 10.1007/s10529-015-1814-4 25792513

[47] Camañes, G., Scalschi, L., Vicedo, B., González-Bosch, C. and García-Agustín, P. (2015) An untargeted global metabolomic analysis reveals the biochemical changes underlying basal resistance and priming in *Solanum lycopersicum*, and identifies 1-methyltryptophan as a metabolite involved in plant responses to *Botrytis cinerea* and *Pseudomonas syringae*. Plant J. 84, 125–139 10.1111/tpj.12964 26270176

[48] Qian, Y., Tan, D.-X., Reiter, R.J. and Shi, H. (2015) Comparative metabolomic analysis highlights the involvement of sugars and glycerol in melatonin-mediated innate immunity against bacterial pathogen in *Arabidopsis*. Sci. Rep. 5, 15815 10.1038/srep15815 26508076 PMC4623600

[49] Suharti, W.S., Nose, A. and Zheng, S.H. (2016) Metabolomic study of two rice lines infected by *Rhizoctonia solani* in negative ion mode by CE/TOF-MS. J. Plant Physiol. 206, 13–24 10.1016/j.jplph.2016.09.004 27688090

[50] Lima, M.R.M., Machado, A.F. and Gubler, W.D. (2017) Metabolomic study of chardonnay grapevines double stressed with esca-associated fungi and drought. Phytopathology 107, 669–680 10.1094/PHYTO-11-16-0410-R 28402211

[51] Bueschl, C., Krska, R., Kluger, B. and Schuhmacher, R. (2013) Isotopic labelingassisted metabolomics using LC-MS. Anal. Bioanal. Chem. 405, 27–33 10.1007/s00216-012-6375-y 23010843 PMC3536965

[52] Chokkathukalam, A., Kim, D.H., Barrett, M.P., Breitling, R. and Creek, D.J. (2014) Stable isotope-labeling studies in metabolomics: new insights into structure and dynamics of metabolic networks. Bioanalysis 6, 511–524 10.4155/bio.13.348 24568354 PMC4048731

[53] You, L., Zhang, B. and Tang, Y.J. (2014) Application of stable isotope-assisted metabolomics for cell metabolism studies. Metabolites 4, 142–165 10.3390/metabo4020142 24957020 PMC4101500

[54] Silva, W.B., Daloso, D.M., Fernie, A.R., Nunes-Nesi, A. and Araujo, W.L. (2016) Can stable isotope mass spectrometry replace radiolabelled approaches in metabolic studies? Plant Sci. 249, 59–69 10.1016/j.plantsci.2016.05.011 27297990

[55] Pang, Q., Zhang, T., Wang, Y., Kong, W., Guan, Q., Yan, X. et al. (2018) Metabolomics of early stage plant cell–microbe interaction using stable isotope labeling. Front. Plant Sci. 9, 760 10.3389/fpls.2018.00760 29922325 PMC5996122

[56] Gulati, S., Ballhausen, M.B., Kulkarni, P., Grosch, R. and Garbeva, P. (2020) A non-invasive soil-based setup to study tomato root volatiles released by healthy and infected roots. Sci. Rep. 10, 1–11 10.1038/s41598-020-69468-z 32728091 PMC7391657

[57] Sun, K., Habteselassie, M.Y., Liu, J., Li, S. and Gao, Y. (2018) Subcellular distribution and biotransformation of phenanthrene in pakchoi after inoculation with endophytic *Pseudomonas* sp. as probed using HRMS coupled with isotope-labeling. Environ. Pollut. 237, 858–867 10.1016/j.envpol.2017.11.039 29150254

[58] Wallace, M.A.G. and McCord, J.P. (2020). High-resolution mass spectrometry. In Breathborne Biomarkers and the Human Volatilome Second edition (Beauchamp, J., Davis, C. and Pleil, J. eds) Elsevier, Amsterdam, Netherlands, pp. 253–270, Elsevier

[59] Oburger, E. and Jones, D.L. (2018) Sampling root exudates–mission impossible? Rhizosphere 6, 116–133 10.1016/j.rhisph.2018.06.004

